# Advanced Functional Materials Research: Paving the Way for Technological Innovation and Beyond

**DOI:** 10.1002/smll.202513910

**Published:** 2025-12-29

**Authors:** Huaping Zhao, Yong Lei

**Affiliations:** ^1^ Fachgebiet Angewandte Nanophysik, Institut für Physik and IMN MacroNano Technische Universität Ilmenau Ilmenau Germany

The evolution of human civilization has always been driven by technological innovation, which in turn fundamentally relies on advancements in materials. From the Stone Age to the Mechanical Age and finally to the Electronic and Information Ages, every leap forward in human progress has stemmed from breakthroughs in materials science and engineering. As we are now transitioning from the Information Age to the Artificial Intelligence Age, increasingly stringent requirements are being placed on the development of advanced functional materials. In particular, materials with exceptional multifunctionality, sustainability, and adaptability to complex environments are becoming particularly important. Meeting these challenges requires continuous exploration in the field of functional materials through the integration of innovative manufacturing methods, advanced characterization techniques, and intelligent computational tools. This integration can deepen our understanding of material properties and help design and develop novel materials, thereby supporting the technological advancements in fields such as optics, electronics, energy, catalysis, biomedicine, and sensors. Therefore, extensive efforts have been devoted to exploring and advancing functional materials to meet the needs of next‐generation technologies and sustainable development. Against this backdrop, international conferences have become important platforms for sharing innovative ideas and promoting collaborations among global researchers in various fields. Through these exchanges, the global materials community can jointly advance research on functional materials and contribute to future technological innovation.

In line with this trend, the AEFM (*Advanced Energy and Functional Materials Research*) 2024 conference was held at the Technische Universität Ilmenau in Germany, from September 30th to October 2nd, 2024, in collaboration with Wiley journals, including *Small, Advanced Energy Materials*, *Energy & Environmental Materials*, and *Carbon Energy*. During the three‐day conference, hundreds of participants from eleven countries shared more than 100 oral and poster presentations, focusing on advanced materials and their applications in fields such as energy, catalysis, environment, optics, and health. The AEFM 2024 conference launched twin issues in *Advanced Energy Materials* and *Small*, respectively, presenting cutting‐edge research on advanced materials for device applications. The published *Advanced Energy Materials* special issue, titled “Advanced Energy Materials Research: Key to Sustainable Energy Future”, covered a broad range of topics in advanced materials for energy conversion and storage applications. This unique special issue of *Small* showcases forty research articles, six review articles, and five perspective articles. These contributions encompass a wide range of research findings in the field of functional materials, many of which stem from international collaborative efforts, showcasing new advances in synthesis, characterization, and applications.

Material research spans multiple levels, from atomic and molecular manipulation to interfacial engineering and structural design, for achieving customized functionalities. By controlling the energy‐level alignment at atomic and molecular levels, these efforts aim to unlock novel quantum transport and spin functions. Kröger et al. elucidated the mechanisms of spin‐excited energy level crossing and anti‐crossing in two exchange‐coupled nickelocenes, providing new insights for designing nanoscale magnetic probes and controlling quantum spin interactions (article number 202412703). Furthermore, his team demonstrated that molecular orbital energies can be tuned near the Fermi level through chemical manipulation, interfacial hybridization, and external field control, thereby effectively modulating the Andreev reflection (article number 202412706).

In addition to atomic‐ and molecular‐level regulation, macroscopic interfacial engineering is another key strategy for improving material properties. Jacobs et al. found that bimetallic interfaces in deposited layers with controlled micromorphology can largely affect wavefront propagation velocity in a vapor‐phase electrodeposited multilayer stacked structure (article number 202501263). In another work, Oschatz explored the interfacial interactions between ionic liquids and microporous carbon materials, revealing that gas adsorption performance can be finely tuned by adjusting the pore size and ionic liquid composition (article number 202501928). In addition, Liu and his co‐workers reported a highly hydrophilic and acidic modified nanocarbon prepared from municipal wastes via a sustainable low‐temperature synthesis route (article number 202412754). Furthermore, Shi's group designed a multifunctional composite coating that combines excellent corrosion resistance, photothermal self‐healing ability, and anti‐biofouling properties (article number 202411729). These studies collectively demonstrate that precise surface chemical modification is crucial for tailoring interfacial properties, thereby enabling targeted applications such as catalytic reactions, gas separation, energy storage, and protective coatings.

Structural design plays a crucial role in enhancing the functionality of materials for optical, electronic, and electrochemical applications. A review by Lei et al. elucidated the structure‐function relationship of advanced materials and provided valuable guidance for the functionalization of structural materials (article number 202412780). Recognizing the importance of structural design, Wang's group designed a 4H‐SiC UV enhanced photodetector based on periodic triangular Al/Al_2_O_3_ core–shell nanoparticle arrays (article number 202502011). The device showed extremely low dark current, high responsivity, and nanosecond response speed. Similarly, Wang et al. constructed a surface‐enhanced Raman scattering microfluidic chip using nanoporous Au decorated with Ag for detecting environmental pollutants (article number 202503894). The group of Wang fabricated a 2D plasmonic lattice based on aluminium nanocone arrays (article number 202412315), which supports narrow‐linewidth resonances and provides directional and polarization‐dependent nanoscale laser at red wavelengths. The work highlights the potential of plasmonic lattices for on‐chip nanoscale lasers. Furthermore, Wang's group designed a quasi‐2D perovskite photonic crystal laser, achieving single‐mode green laser output with a linewidth as narrow as 0.9 nm and an ultralow threshold (article number 202412728). These research advances demonstrate various nanostructuring strategies to enhance light‐matter interactions, thereby promoting the development of integrated photonic and functional materials for applications such as light sources, detection, and environmental sensing.

The rapid growth of electric vehicles (EVs) and residential energy storage systems continues to drive requirements on lithium‐ion batteries (LIBs). Ma's group designed carbon‐coated spherical 2D silicon nanosheets as anode materials to improve the energy density and stability of LIBs (article number 202412705). Yang et al. found that the construction of covalent bonds between black phosphorus and the sp2‐bonded carbon framework can improve electronic conductivity and alleviate volume changes, thereby maintaining the ultrastable cycling performance for LIBs (article number 202504480). Cao studied the reaction mechanism of FeNbO_4_ porous nanofiber and adjusted the working potential window to avoid irreversible reaction, finally extending the cycling lifetime of LIBs (article number 202411792). Compared to LIBs, lithium‐sulfur (Li‐S) batteries have a high theoretical capacity of 1675 mAh g^−1^, but facing challenges of the shuttle effect of lithium polysulfides. A review by Shao summarized the progress in electrospinning‐driven heterostructured cathodes, anodes, and separators, and analysed their working mechanisms for Li‐S batteries (article number 202411838). In another study, they embedded molybdenum carbide within a hierarchical porous carbon nanosheet and used this composite as the separator to improve the energy density and stability of Li‐S batteries (article number 202410907).

Since LIBs are considered the main cause of fatal fire accidents, it is essential to design fail‐safe LIBs to guarantee EVs safety. Sun et al. summarized the safety challenges, failure mechanisms, and targeted solutions for battery safety in EVs (article number 202503406). To address the safety issue, solid‐state lithium batteries emerged due to their inherent safety and high energy density. Wang developed a 3D MOF/polymer fibrous membrane to modify the solvation structure of Li^+^ and reduce interfacial side reactions, thus stabilizing the electrode‐electrolyte interface and presenting extraordinary rate capability and cycling stability (article number 202412494). Additionally, Liu's group proposed a polymer‐in‐salt binder to construct hydrophobic composite sulfide electrolytes with high ionic conductivity, good mechanical properties (article number 202503875). The assembled all‐solid‐state lithium batteries deliver high capacity and stable cycling performance.

Owing to the abundance resources, low cost, and high safety, sodium‐ion batteries (SIBs) have emerged as an attractive candidate for large‐scale energy storage. Cathodes with high voltage have a significant impact on the energy density of SIBs. To guide the design for high‐energy‐density and high‐power‐density cathodes of SIBs, Lei et al. summarized the strategies to promote high‐voltage SIB cathodes, including elemental doping, surface coating, electrolyte optimization, and synthesis methods (article number 202501262). Wu proposed a high‐entropy micro‐doping strategy to prepare O_3_‐type layered oxide cathode (article number 202412023), maintaining phase stability and fast Na diffusion kinetics. Besides the cathode, the anode is also important in battery systems. Lei proposed a co‐precipitation doping strategy to optimize the conductivity of anode materials for improving rate capability (article number 202412449), and found that the iron doping into octahedral sites effectively fastens electron and ion diffusion, achieving superior rate performance. Additionally, Lei and Shao found that binary metal sulfides prepared via a one‐step simultaneous carbonation and sulfidation process maintain the integrity of carbon coating, thus exhibiting accelerated Na^+^ migration kinetics and remarkable cycling performance (article number 202412776). Organic materials with low price, abundant sources, and eco‐compatibility become highly attractive for battery applications. Lei et al. reviewed dissolution challenges of organic molecules in organic sodium‐ion batteries, analysing issues from the aspects of organic electrodes, separators, and electrolytes, proposing mitigation strategies for enhanced electrochemical stability (article number 202412769). Beyond practical experiments, machine learning has become a forward‐looking tool for accelerating the discovery of energy materials. Runge's team has developed a model that captures a detailed and interpretable representation of the material space by applying a unified manifold approximation projection for dimensionality reduction, and accurately predicts the performance of energy material (article number 202412519).

Electrolyte design is equally crucial to battery stability and performance. Balducci proposed dual‐salt electrolytes that exhibit wide and stable potential windows, good transport properties, and low anodic dissolution (article number 202410704). Electrolyte modification allows high‐voltage operation and a homogeneous inorganic‐rich cathode‐electrolyte interphase, achieving high performance for SIBs. With a focus on the limitation of low ionic conductivity of solid‐state electrolytes in the development of solid‐state potassium batteries (SSPBs), Cao et al. summarized ion conduction mechanisms of inorganic and polymer electrolytes, and the corresponding strategies to improve the electrochemical performance for SSPBs (article number 202500762). Aqueous zinc metal batteries have been regarded as one of the potential energy storage systems due to their inherent safety and low cost. However, aqueous electrolytes lead to problems such as uneven zinc dendrite growth, self‐corrosion, and poor low‐temperature adaptability. To this end, Liu proposed a multi‐component hydrogel electrolyte to homogenize Zn^2+^ transfer and disrupt the H‐bond network, thereby stabilizing Zn plating/stripping and maintaining operation under extreme conditions (article number 202501089).

Supercapacitors with high‐power density and ultrastable cycle life are ideal for fast charging‐discharging systems. Liu's team proposed sandwich‐type covalent organic framework (COF) heterojunctions for constructing in‐plane micro‐supercapacitors, which exhibited high energy density and low self‐discharge property (article number 202412642). In another study, Wang et al. designed a nickel sulfoselenide heterostructure array with high conductivity and abundant active sites for potassium‐ion hybrid capacitors, achieving both high power and energy densities (article number 202500411).

Catalytic materials are the foundation of energy conversion technologies, such as water splitting, fuel cells, and electrochemical carbon dioxide (CO_2_) reduction, and their activity and durability directly affect the catalytic efficiency. Carbon dioxide reduction reaction (CO_2_RR) is a sustainable green strategy that converts CO_2_ into valuable fuels and chemicals. Among the wide variety of CO_2_RR electrocatalysts, Cu‐based catalysts are most attractive for converting CO_2_ into multi‐carbon (C_2+_) products. Lv et al. prepared 3D Cu microbuds with polycrystalline characteristics, which enhanced grain boundaries and improved the selectivity of C_2+_ products (article number 202412672). Wang and his co‐workers found that Cu‐based catalysts with bimetallic tandem interfaces can significantly excite Cu^+^ species and enhance the adsorption of key CO_2_RR intermediates, thus suppressing HER and improving CO_2_RR activity (article number 202501125).

Oxygen evolution reaction (OER) and hydrogen evolution reaction (HER) are central to energy conversion and storage technologies. To address the issues of the sluggish OER kinetics and high cost of precious metals OER catalysts, Xing's group reported developed Zr‐doped WRuO_x_ electrocatalyst (article number 202411117), which inhibited over‐oxidation of Ru and the participation of lattice oxygen, and achieved enhanced durability and superior activity under acidic OER conditions. Transition metal materials have also garnered attention as cost‐effective alternatives to precious metals‐based OER catalysts. Xu found that doping Fe in Fe‐Co LDH/MoP heterostructure can accelerate the superexchange interaction between high‐spin Fe (III) and low‐spin Co (II), thus promoting the generation of Co (III) active species and improving OER performance (article number 202505953). Likewise, Yang and his co‐workers reported that sulfur‐rich surfaces in phase‐mixed bi‐NiS_x_ nanoarrays exhibit excellent OER activity (article number 202503194). Magnetoelectrocatalysis is a promising technique for enhancing the electrocatalytic performance of water splitting using static magnetic fields. However, inconsistencies between theoretical explanations and experimental design have hindered its systematic development. In this context, Tsang provided a comprehensive theoretical and methodological framework for the influence of magnetic field on OER, and detailed experimental strategies for improving reproducibility (article number 202500001). Leveraging the understanding of magnetoelectric mechanism, they investigated the Lorentz force and spin polarization effects by adjusting the intensity and orientation, and proved that the magnetic co‐catalyst can amplify the magnetoelectric effect (article number 202412852). For hydrogen evolution, a FeRu alloy electrocatalyst integrated with molybdenum substrates was designed by Chang and Xing, exhibiting high activity in alkaline and seawater conditions (article number 202412729). Shi's group has gone beyond the traditional water splitting reaction and investigated the electrocatalytic oxidation of benzyl alcohol using a bifunctional Co_0.33_Ni_0.67_S_1_‐10c catalyst, which is a sustainable way for producing high‐value fuels. The bifunctional catalyst promotes the hydrogen evolution and maintains a high benzyl alcohol conversion efficiency in alkaline solution (article number 202412734).

Solar‐driven energy conversion technologies offer an environmentally benign and cost‐effective route to produce fuels and chemicals. Cao et al. summarized recent progress in ferrielectric materials for photoelectrochemical water splitting with a focus on using ferrielectric materials to modulate charge separation efficiency and enhance surface reaction kinetics (article number 202412794). Hao found that the optimized band‐matching mechanism of heterojunction interface in CdS/MoS_2_ system promotes the separation of photogenerated charge carriers and exhibits remarkable water reduction performance (article number 202411128). Zhang and Cao explored CO_2_ photoreduction performance of Zn_0.5_Cd_0.5_S QDs/BaTiO_3_ heterojunction by applying an external‐electric‐field to regulate BaTiO_3_ (article number 202412763). Dreßler proposed a multiscale simulation method to achieve near ab initio accuracy and extend the simulation capability to millisecond‐level diffusion timescales, enabling more realistic modelings of transport processes in functional materials (article number 202500931).

Functional materials are widely used in fields of biomedicine, sensors, magnetism, and electromagnetic shielding. Singh proposed a scheme combining 3D microcontact printing and scraping technique to create site‐selective tri‐cultures on both sides of a porous scaffold, thereby enabling the construction of realistic 3D tissue models (article number 202412409). In addition, Schober summarized multiscale engineering in hematopoietic stem cells and brain organoid research, based on two factors: geometric and structural parameters (article number 202504070). For bioanalytical applications, Cao et al. demonstrated that goldbodies based on gold nanoparticles can serve as a stable and efficient alternative to natural antibodies in immunoassays (article number 202412730). Functional materials have driven the development of sensor devices with high sensitivity and stability under extreme conditions. Xu prepared a 2D LaAlO_3_/SrTiO_3_ heterojunction to create an interfacial oxygen vacancy layer, achieving highsensitivity and stable pressure sensor (article number 202412749). Likewise, Wang designed a structurally controllable and robust 4H‐SiC JBS temperature sensor, which exhibits high sensitivity and high linearity in extremely high‐temperature environments (article number 202509679). Progress has also been made in the field of magnetism and electromagnetic shielding. Xu and her colleagues developed a new method to induce room‐temperature ferromagnetism in BaZrO_3_ by supercritical CO_2_ treatment without adding magnetic elements (article number 202411243). Yang and his co‐workers prepared hollow porous Ti_3_C_2_T_x_ MXene films embedded with nickel nanoparticles for efficient electromagnetic shielding (article number 202501928).

This special issue covers a broad range of materials, from microscopic control to macroscopic applications, with the goal of stimulating technological innovation in functional materials. We extend our sincere gratitude to all the participants, including speakers, attendees, and publishers; it is their collective efforts that made the AEFM Conference a great success. We also extend our special thanks to the authors and reviewers for their invaluable contributions to this issue. Last but not least, we sincerely appreciate the strong support and hard work of the editorial team of *Small*, especially Dr. Neville Compton (Editor‐in‐Chief, *Small*), whose efforts ensured the successful publication of this special issue.

## Funding

Chinesisch‐Deutsche Zentrum für Wissenschaftsförderung GZ1579; Deutsche Forschungsgemeinschaft 501766751.

## Conflicts of Interest

The authors declare no conflicts of interest.

